# Inverse treatment planning using deep learning-based organs at risk in radiotherapy for head and neck cancer: a prospective planning study

**DOI:** 10.1007/s00066-026-02519-y

**Published:** 2026-03-30

**Authors:** Tristan Bauer, Oliver Weinhold, Ulrich Schratzenstaller, Andrei Bunea, Joshua Giambattista, Jon Giambattista, Alexandros Papachristofilou, Tobias Finazzi

**Affiliations:** 1 Clinic of Radiotherapy and Radiation Oncology, University Hospital Basel, Basel, Switzerland; 2 Department of Radiation Oncology, BORAD, Bottrop, Germany; 3 Allan Blair Cancer Centre, Regina, Saskatchewan Canada; 4 Limbus AI; now Radformation, Radformation Inc., New York, USA; 5 Department of Radiation Oncology, Cantonal Hospital Baden, Baden, Switzerland

**Keywords:** Head and neck, Radiotherapy, Radiation therapy, Artificial intelligence, Deep learning

## Abstract

**Purpose:**

Radiotherapy (RT) planning for head and neck squamous cell carcinoma (HNSCC) is known to be both challenging and time-consuming. Deep learning (DL)-based auto-segmentation of organs at risk (OARs) may streamline this procedure, although studies have mainly evaluated the geometric accuracy of DL-based contours. Here, we report on a prospective study of inverse treatment planning using DL-based OARs in HNSCC.

**Methods:**

This prospective single-center study enrolled 25 patients undergoing definitive or postoperative (chemo-)radiotherapy for HNSCC. Deep learning-based OAR contours were generated using a commercially available auto-segmentation software and rated independently by four radiation oncologists. Clinical RT plans were compared with plans that were re-optimized based on DL-based OARs, while the clinical target volumes (CTV) and planning target volumes (PTV) were kept unchanged. Dosimetric parameters for CTV/PTV and manually delineated OARs were compared using Wilcoxon’s signed-rank test.

**Results:**

A total of 259 DL-based contours were rated, with mean scores of > 4 (acceptable with corrections) for all structures. For CTV/PTV coverage, the mean dosimetric differences between clinical and DL-based plans were < 1 Gy for all parameters (D95%, D98%, D2%, Dmean). Similarly, differences between doses to “true” (manual) OARs were largely negligible, with one non-critical outlier observed for the spinal cord. All DL-based treatment plans were deemed clinically acceptable after manual review.

**Conclusion:**

Inverse treatment planning using DL-based OARs in head and neck RT is feasible and results in clinically acceptable plans. Larger studies are required to confirm these results and to move further toward fully automated workflows.

## Introduction

Head and neck squamous cell carcinoma (HNSCC) refers to a group of cancers that originate from squamous cells in the head and neck region, including the pharynx, larynx, and oral cavity. With close to 900,000 cases annually, and 450,000 deaths, the global disease burden is substantial. This is particularly the case for patients of lower socioeconomic status, and those in low- and middle-income countries, as consumption of tobacco, alcohol, and betel nut contribute to a growing incidence of HNSCC [1, 2].

More than 60% of patients present with locally advanced (stage III or IV) disease, which can involve advanced local invasion, lymph node metastases, or both [3]. In these patients, radiation therapy (RT) is an essential treatment component, either as definitive concurrent chemoradiotherapy (CRT) or in the adjuvant or palliative setting [4]. RT planning for HNSCC is a complex and time-consuming procedure, which involves manual delineation of target volumes as well as multiple organs at risk (OARs). Automation of these complex procedures is desirable, as this would spare resources, contribute to standardization, enable timely treatment initiation, and facilitate the distribution of modern RT services in underserved areas [5].

Previous studies have shown that automated contouring based on deep learning (DL) algorithms can achieve a high degree of accuracy for OARs in the head and neck region [6]. In addition, automated solutions for generating intensity modulated radiotherapy (IMRT) or volumetric modulated arc therapy (VMAT) treatment plans have been evaluated for head and neck cancer, and this may improve planning quality and efficiency [7, 8]. One limitation, however, is that auto-contouring studies have mostly evaluated the geometric accuracy of OARs when compared to manually generated volumes, e.g., using the Dice similarity coefficient [6]. From a clinical point of view, small volumetric differences may not always be relevant for treatment planning. To evaluate this, we conducted a prospective study of inverse treatment planning using DL-based OAR contours in head and neck cancer.

## Methods

In this academic single-center study, patients undergoing RT for head and neck cancer at the University Hospital of Basel in Basel, Switzerland, were prospectively enrolled in a non-interventional trial. Patients undergoing either definitive or postoperative RT or CRT for head and neck cancer were eligible for inclusion. All patients had provided written content for further use of coded patient data for research purposes. The study was approved by the Ethics Committee of Northwestern and Central Switzerland (BASEC ID 2020-02405). A software for automatic contouring using DL was initially provided as part of an institutional research agreement with Limbus AI (now Radformation; Radformation Inc., New York, NY, USA). No additional funding was received for the planning or conduct of this study.

### Patients

A total of 25 consecutive patients were enrolled in the study between February 2021 and September 2021. The patient characteristics are summarized in Table 1. Patients had a median age of 66 years (range, 45–80) at time of treatment, and most of them were male (92%). Primary tumors were most commonly located in the oropharynx (56%), and most patients had locoregionally advanced tumors (stage III in 24%; stage IVa–IVc in 44% of cases). Definitive CRT was delivered in 7 patients (28%), whereas 18 patients (72%) were treated in the postoperative setting.

**Table 1:** Characteristics of patients enrolled in the prospective planning study.

		Patients (*n* = 25)
Age, median (range), years		66 (45–80)
Sex	Male	23 (92%)
	Female	2 (8%)
Primary tumor site	Oropharynx	14 (56%)
	Hypopharynx	3 (12%)
	Larynx (supraglottic)	2 (8%)
	Oral cavity	2 (8%)
	Salivary glands	1 (4%)
	Unknown (CUP)	3 (12%)
HPV status	Positive	10 (40%)
	Negative	9 (36%)
	Not assessed	6 (24%)
UICC stage (8th ed.)	I	5 (20%)
	II	3 (12%)
	III	6 (24%)
	IVa–IVc	11 (44%)
Setting	Definitive CRT	7 (28%)
	Postoperative RT	18 (72%)
Dose (tumor/tumor bed)	61.05 Gy	7 (28%)
	66 Gy	10 (40%)
	≥ 70 Gy	8 (32%)

*CRT* chemoradiotherapy, *CUP* cancer of unknown primary, *HPV* human papillomavirus, *RT* radiation therapy, *UICC* Union for International Cancer Control

### Treatment characteristics

All patient cases were discussed in our multidisciplinary tumor board prior to any treatment. Delivery of RT was generally performed in accordance with the 2020 Danish Head and Neck Cancer Group (DAHANCA) guidelines [9]. If indicated, concurrent platinum-based chemotherapy was administered by the Department of Medical Oncology.

Radiotherapy was delivered using VMAT with a simultaneous integrated boost (SIB) approach. In the definitive setting, 70 Gy in 35 fractions was delivered to the high-risk clinical target volume (CTV-HR), which included the gross tumor volume (GTV) for the primary tumor (GTV-P) and involved lymph nodes (LN; GTV-N), with a 5-mm isotropic margin (limited by anatomical borders for tumor spread). An intermediate-risk volume (CTV-IR; GTV with 10-mm expansion, limited by anatomical borders) received 61.25 Gy (35 × 1.75 Gy), whereas the elective low-risk region (CTV-LR) received 52.5 Gy (35 × 1.5 Gy). In the postoperative setting, RT was delivered in 33 fractions. A CTV-HR based on the preoperative GTV‑P and/or GTV‑N was only created in cases of close surgical margins (< 2 mm), incomplete resection (R1), or LN extracapsular extension (ECE). This region received 66 Gy (33 × 2 Gy), whereas the CTV-IR (preoperative GTV with 10-mm expansion, limited by anatomical borders) received 61.05 Gy (33 × 1.85 Gy). The elective CTV-LR was treated to 51.15 Gy (33 × 1.55 Gy) in the postoperative setting.

### Radiotherapy planning workflow

Planning computed tomography (CT) imaging was acquired following patient fixation with a thermoplastic five-point mask. Manual delineation of target volumes was performed by experienced radiation oncologists using all available clinical and imaging information. This generally included 18F-fluorodeoxyglucose positron emission computed tomography (FDG-PET/CT) and/or magnetic resonance imaging (MRI). The selection of elective lymph node target volumes was based on primary tumor site and stage, in accordance with expert recommendations [10].

Following manual delineation of the CTVs (as described), an isotropic margin of 5 mm was added to generate the respective planning target volumes (PTVs). Regional OARs were manually delineated by the radiation oncologist. This included the parotid glands, submandibular glands (unless resected), oral cavity, lips, pharyngeal constrictor muscles (PCM), mandible, larynx, thyroid, brachial plexus, and spinal cord.

Following manual delineation, VMAT treatment plans were created by an experienced dosimetrist as per institutional standards. Dose was prescribed to the mean dose of the PTV for the respective dose level, with a goal of ≥ 95% coverage by 95% of the prescription dose (PD), as recommended by ICRU-83 [11]. Institutional OAR constraints were based on DAHANCA recommendations [9]. The main baseline objectives are summarized in Table 2. We performed PCM sparing in accordance with the DARS trial for dysphagia-optimized RT [12]. Additional constraints (e.g., oral cavity Dmean ≤ 30 Gy; thyroid Dmean ≤ 40 Gy) were not used in all patients but applied where clinically indicated. The priorities assigned to PTV coverage and OAR sparing depended on the clinical scenario in each individual case, with tumor coverage typically being prioritized over non-critical OAR constraints. The latter (e.g., for salivary glands) could be exceeded when this was necessary to achieve sufficient target volume coverage. A planning organ at risk volume (PRV) concept was not used.

**Table 2:** Standard (DAHANCA-based) constraints used for treatment planning in head and neck cancer.

Organ at risk		Constraint	Comment
Spinal cord		Dmax ≤ 50 Gy	–
Parotid gland	Bilateral	Dmean ≤ 26 Gy	–
	Contralateral	Dmean ≤ 20 Gy	–
Submandibular	Contralateral	Dmean ≤ 20 Gy	–
Mandible		Dmax ≤ 72 Gy	–
PCM		Dmean ≤ 50 Gy	Only outside of CTV-HR/CTV-IR; not in the case of posterior pharyngeal wall or retropharyngeal lymph node involvement
Larynx	Full	Dmean < 40 Gy	Not in the case of larynx involvement
	Functional	Dmax < 66 Gy	Refers to glottis and arytenoids; not in the case of larynx involvement

Constraints were used as baseline objectives but could be modified and/or exceeded if this was clinically indicated. Constraints for other OARs were used based on clinical demands in each individual case

*CTV-HR* high-risk clinical target volume, *CTV-IR* intermediate-risk clinical target volume, *DAHANCA* Danish Head and Neck Cancer Group, *OAR* organ at risk, *PCM* pharyngeal constrictor muscles

### Analysis of deep learning-based treatment planning

**Fig. 1:**
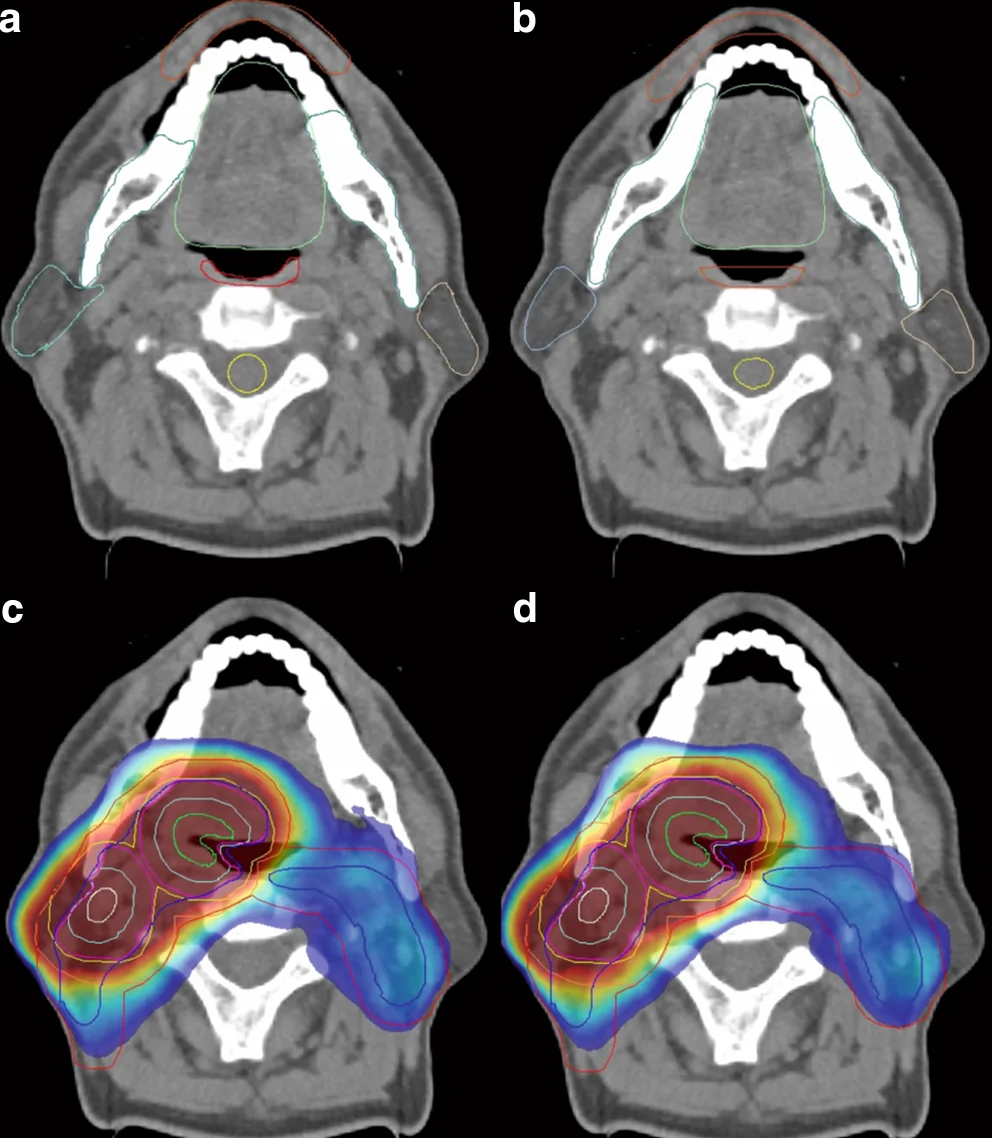
Example of clinical (**a**) and DL-based (**b**) contouring of head and neck OARs (spinal cord, mandible, parotid glands, lips, oral cavity, PCM) in a patient undergoing chemoradiotherapy for right-sided oropharyngeal carcinoma with nodal metastasis. Dose coverage in the clinical plan (**c**) and following re-optimization using DL-based OARs (**d**) is shown below (color wash range: 47.5–66.5 Gy; PTV-LR in red; PTV-IR in orange; PTV-HR in yellow). (Source: University Hospital Basel, used with patient permission). *DL* deep learning, *HR* high-risk, *IR* intermediate-risk, *LR* low-risk, *OARs* organs at risk, *PCM* pharyngeal constrictor muscle, *PTV* planning target volume

In this prospective study, DL-based treatment planning was conducted and evaluated in parallel with the routine planning procedures described previously. First, DL-based segmentation of head and neck OARs (parotids, submandibular glands, oral cavity, lips, mandible, spinal cord, PCM, brachial plexus, larynx and thyroid) was performed using Limbus Contour (v1.3.0 for patients 1–7; upgraded to v1.4.0 for patients 8–25; no systematic difference between versions). An example of DL-based segmentation is shown in Fig. 1. The quality of these DL contours, which were not manually corrected in any way, was rated independently by four radiation oncologists on a scale from 1 (unusable; recontouring from scratch) to 6 (excellent; no or negligible corrections). The DL-based volumes were not made available to the planning radiation oncologists, who first performed the full manual contouring, thereby excluding any influence on the manually generated contours.

Inverse treatment planning was then performed using identical optimization parameters as in the respective clinical plans, but with replacement of the manual OARs by the DL-based volumes. The plans were thus fully re-optimized using the DL-based OARs to evaluate whether clinical objectives would still be met using only DL-based OAR segmentation. The target volumes (CTV, PTV) remained unchanged. Dosimetric parameters were then collected from both clinical (manual) and experimental (DL-based) treatment plans. This included dose coverage (D95%, Dmean) as well as near-minimum (D98%) and near-maximum (D2%) dose for the target volumes, along with selected OAR doses (see Results). For the latter, the manually delineated OARs were used in both clinical and DL-based treatment plans, since these were considered the “ground truth.”

### Statistical analysis

The primary objective of this study was to demonstrate that inverse treatment planning using DL-based OARs is feasible and results in clinically acceptable treatment plans based on target volume coverage (PTV D95% ≥ 95% of PD; primary objective) and OAR doses (see Methods/Table 2; secondary objective). In addition, a pairwise comparison of clinical and DL-based treatment plans was performed using Wilcoxon’s signed-rank test. A value of *p* ≤ 0.05 was considered statistically significant (without Bonferroni correction for multiple comparisons due to the exploratory nature of these tests). As this was a pilot study, a formal power calculation was not conducted. Rather, any outcome would be considered hypothesis-generating and require confirmation in a larger data set. All statistical analyses were performed using RStudio v.2024.12.1+563 (Posit Software, Boston, MA, USA).

## Results

### Rating of deep-learning based contours

A total of 259 DL-based contours were rated by the four radiation oncologists. The results are summarized in Table 3. The overall mean is the average score over all cases, with the lowest and highest mean representing the worst and best rated case(s) for that particular structure. The highest scores were observed for the spinal cord (mean 6.0) and mandible (mean 5.9), whereas the larynx received the lowest scores (mean 4.1).

**Table 3:** Average scores assigned to DL-based OARs by four radiation oncologists, who rated the structures independently. Scale: 1 (unusable, recontouring from scratch); 2 (bad; bulk of structure requires correction); 3 (insufficient; major corrections); 4 (acceptable with corrections); 5 (good; minor corrections); 6 (excellent; no or negligible corrections).

DL-based OAR	Score (1–6)		
	Mean (overall)	Lowest mean	Highest mean
Parotid	5.3	4.3	6.0
Submandibular	4.9	2.5	6.0
Oral cavity	4.9	4.3	5.8
Lips	5.3	3.5	6.0
Mandible	5.9	5.0	6.0
Thyroid	5.2	3.8	6.0
Larynx	4.1	1.8	5.5
Brachial plexus	4.6	3.0	6.0
Spinal cord	6.0	5.8	6.0
PCM	5.3	1.3	6.0

*DL* deep learning, *OARs* organs at risk, *PCM* pharyngeal constrictor muscles

### Evaluation of deep-learning based treatment plans

**Fig. 2:**
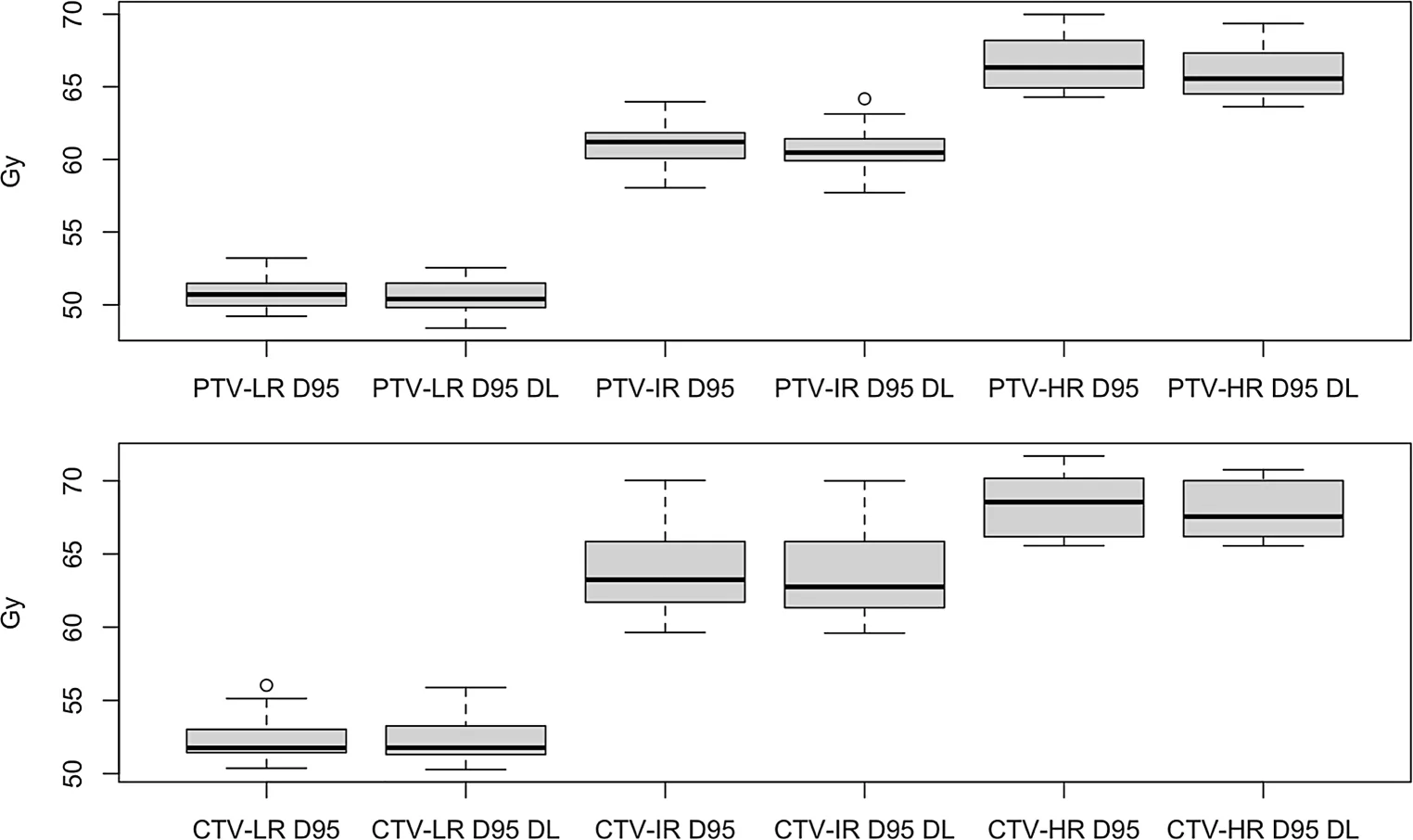
Box plots of PTV and CTV coverage by 95% of the prescribed dose (D95%) in clinical and DL-based treatment plans. Mean differences were < 1 Gy for all parameters. *CTV* clinical target volume, *DL* deep learning, *HR* high-risk, *IR* intermediate-risk, *LR* low-risk, *PTV* planning target volume

Results of target volume coverage in clinical and DL-based treatment plans for the 25 patients are shown in Table 4 and visualized (for D95%) in Fig. 2. For PTV-LR and CTV-LR, the mean D95% was 51.0 Gy (range, 49.2–53.2) and 52.5 Gy (50.4–56.0) in clinical plans, compared to 50.5 Gy (48.4–52.6) and 52.3 Gy (50.3–55.9) in DL-based plans (PTV-LR: *p* < 0.01; CTV-LR: *p* = 0.17). For PTV-IR and CTV-IR, the mean D95% was 61.3 Gy (58.1–64.0) and 64.2 Gy (59.6–70.0) in clinical plans, with respective values of 60.7 Gy (57.7–64.2) and 63.9 Gy (59.6–70.0) in DL-based plans (PTV-IR: *p* < 0.01; CTV-IR: *p* = 0.01). For PTV-HR and CTV-HR (when applied; *n* = 18 patients), the mean D95% was 66.7 Gy (64.3–70.0) and 68.3 Gy (65.6–71.7) in clinical plans, and 66.0 Gy (63.6–69.4) and 68.1 Gy (65.6–70.07) in DL-based plans (PTV-HR: *p* < 0.01; CTV-HR: *p* = 0.12). In terms of percentage, the mean difference in coverage (D95%) by the PD (100%) was ≤ 1% (range, 0–3%) for PTV-LR, PTV-IR, and PTV-HR, and coverage by 95% of PD was > 95% in all plans. Additional dose values are presented in Table 4. Although formal statistical differences were observed for multiple values (except for D2%/near-maximum), the average difference between clinical and DL-based plans was < 1 Gy for all parameters. Target coverage was deemed clinically acceptable in all DL-based treatment plans after manual review by the radiation oncologists.

**Table 4:** Comparison of target volume and OAR doses in clinical and DL-based treatment plans.

**Target volume**	**Parameter**	**Clinical plans**	**DL-based plans**	**Mean ∆ (range)**	***p***
PTV-LR	D95% (Gy)	51.0	50.5	−0.5 (−1.5; 0.7)	*p* < 0.01
PTV-LR	Dmean (Gy)	58.7	58.3	−0.4 (−4.8; 0.5)	*p* < 0.01
PTV-LR	D98% (Gy)	49.9	49.3	−0.7 (−2.0; 1.0)	*p* < 0.01
PTV-LR	D2% (Gy)	69.3	69.3	0.1 (−1.1; 1.2)	*p* = 0.65
PTV-IR	D95% (Gy)	61.3	60.7	−0.5 (−2.0; 0.4)	*p* < 0.01
PTV-IR	Dmean (Gy)	65.7	65.5	−0.2 (−1.2; 0.6)	*p* < 0.01
PTV-IR	D98% (Gy)	60.1	59.5	−0.6 (−2.4; 0.6)	*p* < 0.01
PTV-IR	D2% (Gy)	69.8	69.9	0.1 (−0.8; 1.4)	*p* = 0.29
PTV-HR	D95% (Gy)	66.7	66.0	−0.7 (−2.0; 0.4)	*p* < 0.01
PTV-HR	Dmean (Gy)	69.4	69.2	−0.2 (−1.3; 0.2)	*p* = 0.03
PTV-HR	D98% (Gy)	65.7	64.9	−0.8 (−2.7; 0.5)	*p* < 0.01
PTV-HR	D2% (Gy)	72.1	72.2	0.1 (−0.7; 1.2)	*p* = 0.52
CTV-LR	D95% (Gy)	52.5	52.3	−0.1 (−0.7; 0.7)	*p* = 0.17
CTV-LR	Dmean (Gy)	60.6	60.6	−0.1 (−0.8; 0.9)	*p* = 0.28
CTV-LR	D98% (Gy)	51.9	51.6	−0.2 (−1.1; 0.5)	*p* = 0.02
CTV-IR	D95% (Gy)	64.2	63.9	−0.3 (−1.8; 0.6)	*p* = 0.01
CTV-IR	Dmean (Gy)	67.0	66.9	−0.1 (−0.9; 1.2)	*p* = 0.35
CTV-IR	D98% (Gy)	63.5	63.1	−0.4 (−2.1; 0.5)	*p* < 0.01
CTV-HR	D95% (Gy)	68.3	68.1	−0.3 (−1.4; 0.8)	*p* = 0.12
CTV-HR	Dmean (Gy)	70.2	70.1	−0.1 (−1.3; 0.7)	*p* = 0.98
CTV-HR	D98% (Gy)	67.8	67.5	−0.3 (−1.5, 0.6)	*p* = 0.02
**OAR**	**Parameter**	**Clinical plans**	**DL-based plans**	**Mean ∆ (range)**	***p***
Parotid right	Dmean (Gy)	35.9	36.0	0.1 (−2.4; 4.3)	*p* = 0.77
Parotid right	V30Gy (%)	62.7	63.2	0.5 (−8.4; 10.1)	*p* = 0.63
Parotid right	V5Gy (%)	96.5	97.8	1.4 (−9.0; 39.4)	*p* = 0.90
Parotid left	Dmean (Gy)	34.0	34.1	0.1 (−3.3; 3.8)	*p* = 0.89
Parotid left	V30Gy (%)	55.9	56.1	0.2 (−5.9; 9.2)	*p* = 0.99
Parotid left	V5Gy (%)	93.6	93.1	−0.5 (−41.7; 26.5)	*p* = 0.94
Submandibular right (*n* = 21)	Dmean (Gy)	55.3	55.4	0.1 (−1.9; 2.0)	*p* = 0.79
Submandibular left (*n* = 19)	Dmean (Gy)	49.4	49.8	0.4 (−0.8; 6.5)	*p* = 0.65
Mandible	Dmean (Gy)	40.8	40.7	−0.1 (−2.4; 2.1)	*p* = 0.60
Mandible	V50Gy (%)	33.9	33.3	−0.7 (−6.6; 4.8)	*p* = 0.03
Mandible	D2% (Gy)	64.4	64.4	−0.1 (−1.1; 1.2)	*p* = 0.58
Spinal cord	Dmax (Gy)	42.9	43.8	0.9 (−1.3; 10.4)	*p* = 0.05

Doses are mean values for the 25 patients enrolled in the study, and all OAR doses refer to the manually delineated (i.e., “true”) volumes. Although a statistical difference was observed for various parameters, the absolute differences were overall small

*CTV* clinical target volume, *DL* deep learning, *HR* high-risk, *IR* intermediate-risk, *LR* low-risk, *OAR* organ at risk, *PTV* planning target volume

Doses delivered to the manually delineated (i.e., “true”) OARs are shown in Table 4. Overall, OAR doses were similar in clinical and DL-based treatment plans. Formal statistical differences were observed for the mandible (V50Gy) and spinal cord (Dmax), although these did not appear clinically relevant (Table 4). In one case, the spinal cord Dmax was increased from 34.6 to 45.0 Gy in the DL-based plan. In this case, the clinician had drawn the spinal cord approximately 1–2 mm wider, resulting in a steeper dose gradient toward the OAR than in the DL-based plan. However, this difference of 10.4 Gy was an outlier, as the second-largest difference in spinal cord Dmax was 3.0 Gy (with a mean difference of 0.9 Gy overall). Doses to the salivary glands were largely equal, with notable differences of ± 10% or higher seen in only four cases for parotid V5Gy, and one case for parotid V30Gy. The OAR doses were deemed clinically acceptable in all DL-based treatment plans after manual review.

## Discussion

The head and neck region remains one of the most complex areas for RT planning, considering the proximity of multiple sensitive OARs and the direct impact of suboptimal planning on both tumor control and treatment toxicity [13]. Radiotherapy planning for HNSCC is thus a complex and time-consuming procedure, and delineation of target volumes and OARs may be subject to significant inter-observer variability [14]. The accuracy of DL-based contouring and the feasibility of auto-planning for HNSCC have been previously demonstrated [15–17]. Our small prospective study focused on the use of DL-based OARs for inverse treatment planning and on the ultimate impact on target coverage and dose to the “true” OARs, rather than the geometric accuracy itself.

Although statistical differences were observed for several dosimetric parameters, the absolute impact on CTV and PTV coverage appeared largely negligible, with mean deviations of less than 1 Gy for all parameters (Table 4). Furthermore, mean differences in PD (100%) coverage were ≤ 1% for all dose levels. For reference, common action levels for adaptive re-planning in HNSCC can be considered in the range of 5%, and typically for 95% (rather than 100%) of PD [18]. Although our small study cannot prove statistical non-inferiority of DL-based plans, it does appear unlikely that the differences observed would approach this level of clinical relevance. Similarly, doses to the “true” OARs remained largely stable when RT plans were re-optimized using the DL-based OARs. However, one case with an increase in spinal cord Dmax of approximately 10 Gy serves as a reminder that even small volumetric differences can have an impact, particularly when target volumes and critical OARs are closely related, which is frequently the case in HNSCC. Despite these caveats, our experience demonstrates that inverse treatment planning using DL-generated OARs is feasible and results in clinically acceptable plans. Although geometric accuracy was not the focus of this work, our expert rating also showed that clinicians were overall satisfied with the volumes, with mean scores > 4 (“acceptable with corrections”) for all structures. Of note, the lowest rating scores were given in cases where metal artifacts were present in the CT.

Our small prospective study adds to a body of literature exploring the use of DL for delineation and planning of RT of HNSCC. For the auto-delineation software used in this work, a study previously demonstrated that DL-based head and neck OARs were acceptable based on the Dice similarity coefficient (DSC) and 95% Hausdorff distance, which are similarity measures used to compare automated vs. manual contours [19]. A retrospective study in 10 patients with HNSCC performed recalculation (but not re-optimization) on DL-based OARs, which were generated using either Limbus AI (now Radformation) or AI Rad Companion (Siemens Healthineers, Erlangen, Germany). The report was limited to the brainstem, spinal cord, and parotids, demonstrating median dose–volume histogram (DVH) differences of up to ± 7%, which was not statistically significant [20]. Other studies have explored automation of the treatment planning (rather than contouring) procedure, or focused on more challenging volumes, such as the head and neck lymph node levels [21–23]. In general, these studies demonstrate that DL can be used to generate clinically acceptable results for RT planning in HNSCC.

### Limitations

Our study has several limitations. First, our analysis was limited to a selection of OARs, based on our standard DVH evaluation approach at the time. Although all clinical volumes were delineated by an experienced radiation oncologist, these manually delineated “ground-truth” OARs were not independently rated (e.g., using the same six-point scale), and no quality criteria for these volumes were defined. It can therefore not be excluded that manual corrections of either clinical or DL-based OARs could alter planning outcomes. Furthermore, we did not measure or define how different scores would translate into clinical editing times for the DL-based OARs. Since the conduct of this study in 2021, the auto-segmentation software used has undergone several important updates. In line with other commercially available systems, this included marked improvements in performance, which we observed for various OARs as well as DL-based lymph node CTVs, which were not used in this study. Additional studies could thus include these updated structures, and aim for a fully automated planning workflow, which is a topic of ongoing research [24, 25]. Finally, despite the prospective design, we did not conduct a formal power calculation, and our results are to be considered hypothesis-generating at this stage.

## Conclusion

In conclusion, our small prospective study demonstrated that inverse treatment planning using deep learning (DL)-based organs at risk (OARs) in head and neck radiotherapy (RT) was feasible, and that resulting differences in target volume and OAR doses were small. This outcome may indicate that treatment planning for head and neck squamous cell carcinoma (HNSCC) can be performed using DL-based OARs without manual correction, although this hypothesis requires confirmation in a larger study. The International Atomic Energy Agency (IAEA) has recently launched a coordinated research project to study DL-assisted contouring of head and neck tumors, following a previous project focusing on OARs [26, 27]. These projects emphasize the potential of DL tools to improve the quality of RT for HNSCC on a global level, including in low- and middle-income countries.

## Data Availability

The dataset generated during the current study is available from the corresponding author upon reasonable request.
